# Evaluation of GMI and PMI diffeomorphic‐based demons algorithms for aligning PET and CT Images

**DOI:** 10.1120/jacmp.v16i4.5148

**Published:** 2015-07-08

**Authors:** Juan Yang, Hongjun Wang, You Zhang, Yong Yin

**Affiliations:** ^1^ School of Information Science and Engineering Shandong University Jinan Shandong China; ^2^ Medical Physics Program Duke University, Duke University Medical Center Durham NC USA; ^3^ Department of Radiation Oncology Shandong Cancer Hospital and Institute Jinan Shandong China

**Keywords:** deformable image registration, PET/CT, mutual information, demons algorithm, multimodal

## Abstract

Fusion of anatomic information in computed tomography (CT) and functional information in F18‐FDG positron emission tomography (PET) is crucial for accurate differentiation of tumor from benign masses, designing radiotherapy treatment plan and staging of cancer. Although current PET and CT images can be acquired from combined F18‐FDG PET/CT scanner, the two acquisitions are scanned separately and take a long time, which may induce potential positional errors in global and local caused by respiratory motion or organ peristalsis. So registration (alignment) of whole‐body PET and CT images is a prerequisite for their meaningful fusion. The purpose of this study was to assess the performance of two multimodal registration algorithms for aligning PET and CT images. The proposed gradient of mutual information (GMI)‐based demons algorithm, which incorporated the GMI between two images as an external force to facilitate the alignment, was compared with the point‐wise mutual information (PMI) diffeomorphic‐based demons algorithm whose external force was modified by replacing the image intensity difference in diffeomorphic demons algorithm with the PMI to make it appropriate for multimodal image registration. Eight patients with esophageal cancer(s) were enrolled in this IRB‐approved study. Whole‐body PET and CT images were acquired from a combined F18‐FDG PET/CT scanner for each patient. The modified Hausdorff distance ($d_{\text{MH}}$) was used to evaluate the registration accuracy of the two algorithms. Of all patients, the mean values and standard deviations (SDs) of $d_{\text{MH}}$ were 6.65 (± 1.90) voxels and 6.01 (± 1.90) after the GMI‐based demons and the PMI diffeomorphic‐based demons registration algorithms respectively. Preliminary results on oncological patients showed that the respiratory motion and organ peristalsis in PET/CT esophageal images could not be neglected, although a combined F18‐FDG PET/CT scanner was used for image acquisition. The PMI diffeomorphic‐based demons algorithm was more accurate than the GMI‐based demons algorithm in registering PET/CT esophageal images.

PACS numbers: 87.57.nj, 87.57. Q‐, 87.57.uk

## I. INTRODUCTION

Multimodal image registration and fusion of PET and CT play an important role in clinic because they allow accurate localization of tumors, shown straightforwardly by PET, with respect to detailed anatomic information by CT.^1^, ^2^, ^3^ Furthermore, the precise identification and localization of potential tumors with respect to CT can ensure the delivery of highly conformal doses to the tumors^(4)^ without harming surrounding healthy tissues and organs.^(5)^ Fused PET/CT images have also been used for cancer staging and recurrent tumor detection.^4^, ^6^ The rapid emergence of combined PET/CT scanners in recent years confirms their clinical values in providing complementary information from these two modalities.

Registration of whole‐body PET and CT images is a foundation for the fusion of the two modalities; however, the registration could only be performed manually or using software before the advent of combined PET/CT scanners. The manual registration approaches are slow and tedious and mostly limited to rigid registration. Several software algorithms achieving nonrigid image registration have been reported.^6^, ^7^, ^8^ However, the combined PET/CT scanners have simplified multimodal image registration by performing the PET and CT scans successively with minimized patient movement and then appropriately moving one scan with respect to another. Although the combined scanners have decreased the overall scan time up to 40% by using the CT scan for PET attenuation correction,^(3)^ the registration mode remains rigid, which is not appropriate for correcting the misalignment between PET and CT caused by nonrigid motions of organs. Positional errors in global and local between PET and CT images are unavoidable due to different breathing patterns of patients during multimodal image acquisitions. For example, CT scan is generally performed with breath‐hold or shallow breathing, whereas PET scan may be performed with tidal breathing. Therefore, deformable image registration is required to correct the local misalignment.

For multimodal images, the most popular deformable image registration method was mutual information (MI)‐based algorithm, which has been confirmed as the basic method to align PET and CT images.^9^, ^10^, ^11^ Jan et al.^(12)^ used a rigid transformation to automatically register whole‐body PET‐CT‐SPECT; however, the local nonrigid deformation could not be solved. Castillo et al.^(13)^ presented a new 3D elastic transformation algorithm based on normalized mutual information (NMI) to automatically align PET and CT images, which showed better performance compared with rigid transformation. Suh et al.^(14)^ proposed a novel deformable registration method based on a weighted demons algorithm to register whole‐body rat CT and PET images, and the maximum likelihood Hausdorff distance was used as a similarity measure. Preliminary results demonstrated the high efficiency of this algorithm compared with traditional demons algorithm and NMI‐based nonrigid free‐form deformation (FFD) method. The traditional demons algorithm^(15)^ is a popular and widely used registration method, from which many variations have been derived for monomodal or multimodal image registration. Vercauteran et al.^(16)^ proposed a nonparametric diffeomorphic image registration algorithm based on the classic demons algorithm, and adapted the optimization procedure underlying the demons algorithm to a space of diffeomorphic transformation. This algorithm showed computational efficiency compared with many other diffeomorphic registration methods by replacing an addition of displacement fields with only a few compositions. However, the main drawback of this method was that it could not be used for multimodal images registration. Lu et al.^(17)^ presented a variational approach based on the diffeomorphic demons for multimodal image registration. The point‐wise mutual information (PMI) was employed to replace the standard demons similarity metric (image intensity difference) in the energy function. The accuracy of this algorithm was evaluated by comparing against free‐form deformation (FFD) approach in aligning T1 and T2 brain MR images. Another extended demons algorithm based on gradient of mutual information (GMI) was proposed by Jin et al.^(18)^ By adding an additional external force based on the GMI between two images, the algorithm could be used for processing multimodal images registration. The feasibility of the algorithm was evaluated in 10 esophageal cancer patients by using the modified Hausdorff distance as the similarity metric. Although initial patient results were reported in the pilot study by Jin and colleagues since it included a limited number of patients and assessed only patients with esophageal cancers, the preliminary results have shown efficiency and efficacy of this method used for PET/CT registration.

In our study which aims to perform accurate lesion positioning by incorporating the functional information of PET and the anatomical information of CT, we designed a multiresolution strategy to evaluate and compare two multimodel registration algorithms, named as PMI diffeomorphic‐based demons algorithm and GMI‐based demons algorithm, respectively. Eight patients with esophageal cancer(s) were enrolled in this IRB‐approved study. In addition, here we want to clarify that the patients used in our study are different from patients used in the study by Jin and colleagues, which can potentially ensure the universality and the fairness of comparison and evaluation. Whole body PET and CT images were acquired from a combined F18‐FDG PET/CT scanner for each patient. PET and CT images were down‐sampled by a factor of 2 for three times to reduce the computational time. A rigid registration method was used to preprocess the two images to minimize global misalignment. Then the GMI‐based demons algorithm and the PMI diffeomorphic‐based demons algorithm were used to further register PET and CT images to correct local misalignment in a multiresolution framework. Modified Hausdorff distance was used as the similarity metric to quantitatively evaluate the accuracy of the two algorithms. Preliminary results on oncological patients demonstrated that the PMI diffeomorphic‐based demons algorithm was a superior registration method for PET and CT images.

## II. MATERIALS AND METHODS

### A. Patient cohort and imaging study

Eight patients (four male, four female, mean age of 65.3 yrs) with esophageal cancer(s) were enrolled in this prospective study, which was approved by the Institutional Review Board of Shandong Cancer Hospital and Institute, and all the patients provided written informed consents. All PET/CT scans were obtained prior to the planning CT scan as part of the routine diagnostic protocol for esophageal cancer. All patients were positioned head‐first supine with the arms raised above the head. Vacuum bags were used to immobilize the patients during image acquisition.

All PET and CT scans were performed on a F18‐FDG PET/CT scanner (Discovery LS; GE Healthcare, Waukesha, WI) about 60 min after 370 MBq (10mCi) of F18‐FDG intravenous injection. The PET scans were obtained from head to thigh for 5 min per field of view covering longitudinal distance of 14.5 cm, and the slice thickness of each axial image was 4.5 mm. Four‐slice helical CT acquisition was acquired, followed by full‐ring dedicated PET scan with the same axial range. Both CT and PET scans were obtained during free breathing. CT‐derived attenuation correction was performed for PET reconstruction using ordered‐subset expectation maximization (OSEM) software. The in‐plane resolution of PET was 3.91×3.91 mm, with reconstruction matrix of 128×128. Imaging parameters for CT scans were as follows: 120 kV, 90 mA, a pitch of 6:1, gantry rotation of 0.8 sec per cycle, reconstruction matrix of 512×512, field of view of 450~500 mm, in‐plane resolution of 0.98×0.98 mm. The attenuation‐corrected PET images, CT images, and fused PET/CT images were available in axial, coronal, and sagittal views, using the manufacturer's review station (Xeleris; GE Healthcare).

### B. Classical “demons” algorithm

The classical ‘demons’ algorithm, proposed by Thirion,^(15)^ used gradient information from a static reference image to determine the ‘demons’ force to deform the target image. However, this approach may not be sufficient when the gradient of the reference image is small. For a fixed image $I_{0}$ and a moving image $I_{1}$, the deformation field $\vec{u}$ between two images was calculated as:

(1) $$\vec{u}=\frac{(i_{1}-i_{0})\times {\vec{\nabla }}_{i_{0}}}{{\left\|{\vec{\nabla }}_{i_{0}}\right\|}^{2}+\frac{{\left(i_{1}-i_{0}\right)}^{2}}{k^{2}}}$$

where parameters $i_{1}$ and $i_{0}$ denote the gray scale of corresponding pixels between floating image $I_{1}$ and reference image $I_{0}$, respectively; $\vec{\nabla }$ is the gradient of the images; *k* is a normalization coefficient to compensate the misalignment between the two images.

The purpose of registration is to find out the offset from the floating image to the reference image at each coordinate. As aforementioned, the demons algorithm aligns images depending on the external and internal forces, which indicate the difference of grayscale between the two images and the gradient of the reference image. So it is easy to cause misalignment due to the uncertainty of the movement directions of pixels and the deformation direction of the floating image, when $\left\|\vec{\nabla }i_{0}\right\|\to 0$. Also it can hardly perform accurate transformation between multimodal images, due to the greatly varied grayscale distributions.

### C. Gradient of mutual information (GMI)‐based demons algorithm

As a result of the drawbacks of traditional demons algorithm, Jin et al.^(18)^ proposed a variation by combining the mutual information method to align multimodal images. An additional external force based on the GMI between two images was added to the classical demons algorithm. The iterated updated offset calculated by the GMI‐based demons algorithm was formulated as

(2) $${\vec{u}}_{n+1}=G_{\sigma }*\left({\vec{u}}_{n}+\frac{(i_{1}-i_{0})\times {\vec{\nabla }}_{i_{0}}}{{\left\|{\vec{\nabla }}_{i_{0}}\right\|}^{2}+\frac{{\left(i_{1}-i_{0}\right)}^{2}}{k^{2}}}+\alpha \vec{\nabla }MI({\vec{u}}_{n})\right)$$

where the parameter *Gσ* is the Gaussian filter used for smoothing the offset between iterations to regularize the deformation. For a specific pixel $P\vec{\nabla }\text{MI}({\vec{u}}_{n})$ represents the GMI between two images for current transformation, which is defined as the derivative of mutual information to current spatial displacement vector. Parameter α represents the weighting of additional external force. The authors compared the GMI‐based demons algorithm with the most commonly used multimodal registration algorithm based on mutual information in 10 oncological patients, and concluded that the GMI‐based demons algorithm could perform better registration for PET and CT than the MI based method, with the ratio difference ranging from 2.17% to 10.26%.

### D. Point‐wise mutual information (PMI) diffeomorphic‐based demons algorithm

Given a reference image R, a floating image F, and a transformation field s, the demons algorithm can be described by the energy function with respect to the update field $\vec{u}$ which is calculated as

(3) $$E_{S}(\vec{u})={\left\|R-F\circ (s+\vec{u})\right\|}^{2}+\frac{\sigma_{i}^{2}}{\sigma_{x}^{2}}{\left\|\vec{u}\right\|}^{2}$$

A diffeomorphic extension of traditional demons aiming at solving the drawbacks of inability to generate an invertible output transform was proposed by Vercauteran et al.^(16)^ In diffeomorphic demons framework the update was done through the exponential map on the Lie group, which was described as follows

(4) $$s=s\circ \text{exp}(\vec{u})$$

Although this extension has been proved to be a robust and efficient method for intensity‐based image registration, it could not be used to deal with multimodal images. Lu et al.^(17)^ modified the external forces by replacing the image intensity difference in Eq. (4) with the point‐wise mutual information (PMI) to make it appropriate for multimodal images registration. The global mutual information was described as

(5) $$MI=\frac{1}{N}\sum_{x}S_{MI(x)}$$

where *x* denoted the pixel in image, and *N* was the number of pixels in the overlapping area between two images. $S_{MI(x)}=\text{log}(\frac{p(i_{R}(x),i_{F}(x))}{p(i_{R}(x))p(i_{F}(x))})$, with the parameters $i_{R}$ and $i_{F}$ indicating the image intensities of image *R* and *F*, the energy function would then become

(6) $$E_{s}(\vec{u})=\text{log}(\frac{p(i_{R},i_{F\circ }(S\circ \text{exp}(\vec{u})))}{p(i_{R})p(i_{F\circ }(S\circ \text{exp}(\vec{u})))})$$

A larger mutual information value indicates better alignment, and maximization of the energy is required instead of minimization as in the traditional diffeomorphic demons algorithm. We could simply use the gradient of the PMI as the external force when E reached to the maximum, satisfying $\nabla E(\vec{u})=0$. The forward and reverse forces^(19)^ were utilized to make the optimization more accurate and robust.

The forward force $F_{f}$ is defined as the gradient of the PMI with respect to the reference image. It moves the fixed image to better match the floating image,

(7) $$F_{f}=\frac{\partial }{\partial \in }{|}_{\in =0}S_{MI}(i_{R}(x+\in ),i_{F\circ s}(x))$$

while the reverse force $F_{r}$, which tends to align the points in the moving image with respect to the fixed image, is defined as:

(8) $$F_{f}(x)=\frac{\partial }{\partial \in }{|}_{\in =0}S_{MI}(i_{R}(x),i_{F\circ s}(x+\in ))$$

The update field can finally be defined as:

(9) $$\vec{u}=K_{E}(F_{f}-F_{r})$$

where the coefficient $K_{E}$ indicates the update step length which controls the optimization speed.

### E. Multiresolution strategy and parameters setting

**Figure 1:**
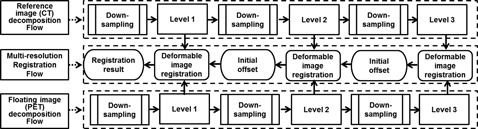
Workflow of multiresolution strategy used in this study. The original PET and CT images were down‐sampled into three resolution levels, and multiresolution registration was performed from the coarsest level to the finest level, consisting 20 iterations each. For the floating image (PET image), the initial offset of each pixel was set to 0. Then the PET and CT images were down‐sampled successively, forming two pyramids. The first alignment of PET and CT was performed using the proposed registration algorithms at the third level with the least image detail, which was served as the coarsest level. After 20 iterations, the current obtained offset of each pixel could be calculated by the registration algorithm and then treated as the initialization for the higher resolution. After all the resolution levels were accomplished, we can achieve precise registration of PET and CT images by applying the final deformation vector field to the floating image (PET).

For both algorithms, a multiresolution strategy was adopted in this study to accelerate registration speed and avoid local extremum. Within a predefined number of iterations, the registration was performed from the coarsest resolution level to the finest resolution level. The reference CT and the floating PET were down‐sampled by a factor of 2 in three dimensions, so eight times fewer voxels of the original data were generated after three times of down‐sampling. This could largely reduce the computational time. The down‐sampled images were then registered in the multiresolution framework shown in Fig. 1. The multiresolution registration was performed from the coarsest level to the finest level, consisting of 20 iterations each. For the floating image (PET image), the initial offset of each pixel was set to 0. Then the PET and CT images were down‐sampled successively, forming two pyramids. The alignment of PET and CT was performed using the proposed registration algorithms, beginning with the third level with the least image detail. After 20 times of iteration, the current obtained offset of each pixel could be calculated by the registration algorithm and was used as the initial value for the higher resolution.

After all the resolution levels were accomplished, the wrapped image was up‐sampled so that it had the same size as the reference image. By applying the final deformation vector field to the floating image (PET), we could achieve precise registration between PET and CT images.

In addition, for the update field regularization, we used a Gaussian kernel with a sigma σ=3 and for deformation field Gaussian regularization, a Gaussian kernel with a sigma σ=2 was used. The GMI‐based demons parameter α was set to 1, and the PMI diffeomorphic‐based demons coefficient $k_{E}$ was set to 5 at the beginning of optimization and decreased proportionally during the iterations to facilitate convergence.

### F. Evaluation and comparison of deformable registration algorithms

Hausdorff distance, which measures the distance of two subsets from each other and is well known as an efficient similarity criterion for low‐level object or image comparison in computer vision, was used as the similarity metric to evaluate the registration performance of the two presented algorithms in our study. This similarity measure uses a set of points extracted by an edge operator.^20^, ^21^ In this paper, we used a three‐dimensional version of modified Hausdorff distance using Canny edge‐detector with a Gaussian smoothing kernel with σ=2. This automated edge‐detection algorithm has been successfully used by Suh et al.,^(14)^ who indicated that the modified Hausdorff distance similarity measure was robust in the presence of outliers and occlusions, and can be used to compare the similarity of PET‐CT images, even though the edges are not fully generated from the PET images. So no extra human observer was enrolled to check their consistency.

Given two sets of edge positions X and Y in two three‐dimensional images with size of I and J, the Hausdorff distance is defined as:

(10) $$d_{H}(X,Y)=\text{max}(d(X,Y),d(Y,X))$$

where *d*(*X,Y*) and *d*(*Y, X*) are the Hausdorff distances of *x* α*r y* and *y* α*r X*, respectively, and are defined as

(11) $$d(X,Y)=\underset{x\in X}{\text{max}}d(x,Y)=\underset{x_{i}\in X}{\text{max}}\underset{y_{i}\in Y}{\text{min}}\left\|x_{i}-y_{i}\right\|$$

(12) $$d(Y,X)=\underset{y\in Y}{\text{max}}d(y,X)=\underset{y_{i}\in Y}{\text{max}}\underset{x_{i}\in X}{\text{min}}\left\|y_{i}-x_{i}\right\|$$

where ‖⋅‖ represents the calculation of distance between two points, and here we restrict it being the Euclidean distance metric.

Although the traditional Hausdorff distance is easy to calculate, it is sensitive to image noise. The modified Hausdorff distance proposed by Dubuisson et al.^(21)^ which was used in this study can avoid the deviation caused by the interference of noisy pixels. The modified Hausdorff distance was defined as:

(13) $$d_{MH}(X,Y)=\frac{1}{I}\sum_{x\in X}d(x,Y)$$

where *d(x,Y)* is the minimum corresponding distance at point, and *I* is the size of the set *X*.

There are many edges from the CT image due to high resolution of anatomical structures. However, there are comparatively simple edge lines in the edge image of the PET due to the poor image quality. The number of feature points identified by the edge‐detection method is patient‐specific and depends on image quality. In this study, we calculated the modified Hausdorff distance values $\left(d_{\text{MH}‐b}\right)$ between the reference image (CT) and the floating image (PET) before registration. Then the modified Hausdorff distance values ($d_{\text{MH}‐r}$) between the reference image (CT) and the floating image after rigid registration was calculated. Further, we calculated the modified Hausdorff distance values between the reference image (CT) and the deformed floating image after using the GMI‐based demons ($d_{\text{MH}–G}$) and the PMI diffeomorphic‐based demons ($d_{\text{MH}–P}$). The difference values between $d_{\text{MH}–r}$ and $d_{\text{MH}–b}$ (D1), and difference values (D2) between $d_{\text{MH}–G}$ and $d_{\text{MH}–P}$ were also calculated.

## III. RESULTS

Preliminary results of PET and CT images registration on a total of eight clinically acquired whole‐body PET/CT image pairs from esophageal cancer patients were reported. The image data were collected by a physician who was not familiar with PET and CT registration algorithm, only on the basis of available multimodal images without any screening criteria.

**Figure 2:**
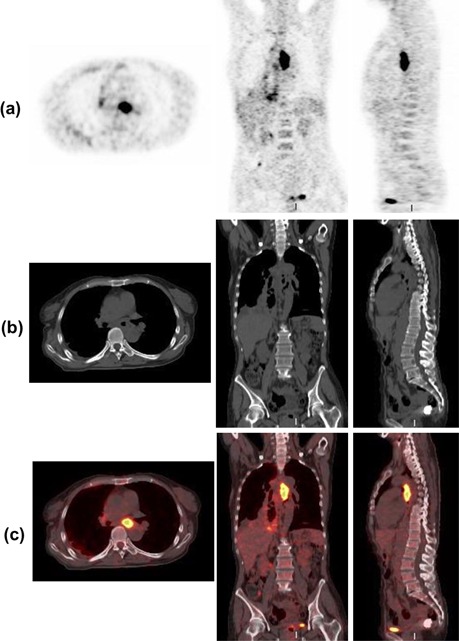
Example of PET and CT images: (a) PET; (b) CT; (c) PET/CT fused.

PET and CT images for a representative patient are shown in Fig. 2. Figure 2 shows PET (a), CT (b), and fusion (c) of PET and CT, respectively. Tumors in PET and fusion images were highly visible due to the high metabolic characteristic of lesions in F18‐FDG PET images.

**Figure 3:**
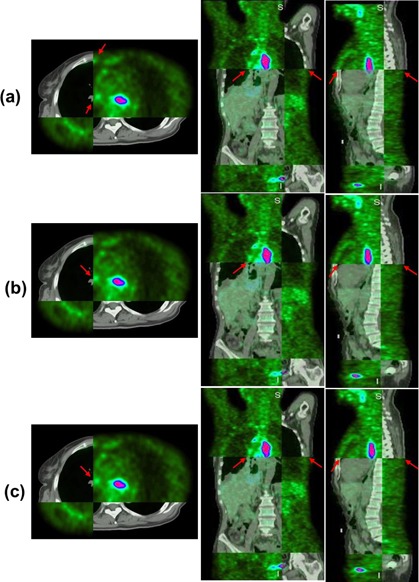
Example of registration results between CT and PET images from Patient #1: (a) PET/CT fused after rigid registration; (b) PET/CT fused after using the GMI‐based demons algorithm; (c) PET/CT fused after the PMI diffeomorphic‐based demons algorithm. The highlighted areas in PET images represented the lesions. Obvious global and local misalignment between PET and CT (indicted by red arrows) could be detected before registration. After using the GMI‐based demons algorithm, the misalignment was greatly corrected, except for several small errors (indicted by red arrows). After using the PMI diffeomorphic‐based demons algorithm, PET and CT images registered each other very well.

In current clinic, positioning tumor target including GTV (gross tumor target) and PTV (planning target volume) in CT images is usually performed based on PET images. Figure 3 shows the registration results of the representative patient in axial, coronal, and sagittal planes. As shown in Fig. 3(a), misalignment between PET and CT (indicted by red arrows) were obvious after rigid registration, which would undoubtedly affect the accuracy of treatment planning and radiotherapy outcome. Through registration of PET/CT, we can accurately position the tumor volume and reduce target area and extended range of tumor volume independently, which ensures high dose of radiation within tumor volume and low dose within adjacent organs and tissues, and achieves the purpose of precise radiotherapy. The highlighted areas in PET images represented the lesions. As shown in Fig. 3(b), after using the GMI‐based demons algorithm, the misalignment was greatly corrected, except for several small errors (indicted by red arrows). As shown in Fig. 3(c), after using the PMI diffeomorphic‐based demons algorithm, PET and CT images were registered each other very well compared with those in Fig. 3(b) identified by an experienced physician, which was helpful for physicians to identify tumor target volume based on the fused PET/CT image, and for physicists to develop accurate radiation treatment planning. After PET and CT images registration, tumor target volume could be accurately positioned which ensured high dose within tumor target and reduced radiation hazard to surrounding organs.

**Figure 4:**
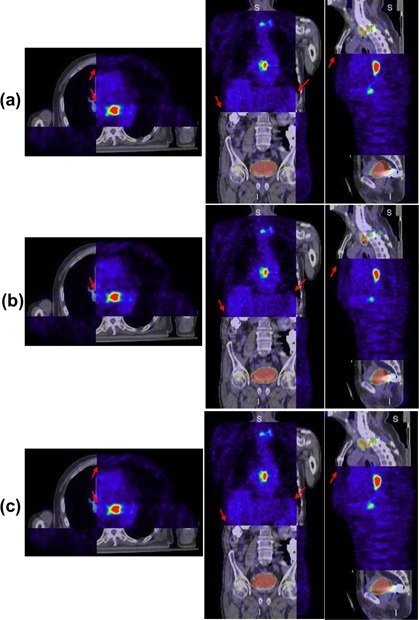
Example of registration results between CT and PET images from Patient #2: (a) PET/CT fused after rigid registration; (b) PET/CT fused after using the GMI‐based demons algorithm; (c) PET/CT fused after the PMI diffeomorphic‐based demons algorithm. The highlighted areas in PET images represented the lesions. Obvious global and local misalignment between PET and CT (indicted by red arrows) could be found before registration. After using the GMI‐based demons algorithm, the misalignment was greatly corrected, except for several small errors (indicted by red arrows). After using the PMI diffeomorphic‐based demons algorithm, PET and CT images registered each other very well.

Figure 4 shows another clinical example illustrating the registration results of the GMI‐based demons algorithm (Fig. 4(b)) and the PMI diffeomorphic‐based demons algorithm (Fig. 4(c)). As shown in Fig. 4(a), we found that the misalignment indicated by the red arrows in axial, coronal, and sagittal planes were obvious after rigid registration. After using the GMI‐based demons algorithm, the misalignment was greatly corrected, except for several small errors (indicated by the red arrows). After using the PMI diffeomorphic‐based demons algorithm, the PET and CT images were registered each other very well (indicated by the red arrows).

Table 1 summarizes the measurement results of the modified Hausdorff distance measurements before and after the GMI‐based demons and the PMI diffeomorphic‐based demons registration algorithms in all eight esophageal cancer patients. Table 1 shows the comparison of spatial consistency of two images before registration and after rigid, GMI‐based demons and PMI diffeomorphic‐based demons registration. And the unit for the measured values is voxels, which shows the comparison of spatial consistency of two images in voxels before and after registration. On average, the mean value and standard deviation (SD) of $d_{\text{MH}–b}$ was 7.96 (± 1.89). The mean (± SD) value of $d_{\text{MH}–r}$ was 7.40 (± 1.72) which was reduced compared with that of before registration, with the mean difference value of 0.56 (± 0.38). The mean values (7.96 and 7.40) are calculated by averaging the measured values before and after rigid registration from 10 patients. The standard deviation values (1.89 and 1.72) are to measure dispersion degree of data distribution, that is, the degree of deviating from the mean values. The smaller the standard deviation, the less these values deviating from the average. The mean (± SD) values of $d_{\text{MH}–G}$ and $d_{\text{MH}–P}$ were 6.65 (± 1.90) and 6.01 (± 1.90) after the GMI‐based demons algorithm and the PMI diffeomorphic‐based demons algorithm, respectively. It was illustrated that the $d_{\text{MH}}$ values after deformable registration were reduced compared with those after rigid registration, and the $d_{\text{MH}}$ values after the PMI diffeomorphic‐based demons algorithm were less than those after the GMI‐based demons algorithm, with the mean difference value of 0.65 (± 0.38), which demonstrated that the PMI diffeomorphic‐based demons algorithm performed more accurate registration in aligning esophageal PET/CT images than the GMI‐based demons algorithm.

**Table 1:** Measurement results of modified Hausdorff distance (M‐HD) (Unit: voxels) before and after using the GMI‐based and PMI diffeomorphic‐based demons algorithms in eight esophageal patients

				*Deformable Registration*		
*Patient*	*Before Registration* ${\left(d_{MH-b}\right)}^{a}$	*After Global Registration* ${\left(d_{MH-r}\right)}^{b}$	*Difference* ${\left(DI\right)}^{c}$	*After GMI‐based Demons* ${\left(d_{MH-G}\right)}^{d}$	*After PMI Diffeomorphic‐based Demons* ${\left(d_{MH-P}\right)}^{e}$	*Difference* ${\left(D2\right)}^{f}$
1	8.87	7.56	1.31	6.77	6.27	0.5
2	5.66	5.35	0.31	4.56	4.06	0.5
3	9.65	9.06	0.59	8.88	8.02	0.86
4	6.86	6.06	0.80	5.10	4.20	0.9
5	7.56	7.28	0.28	6.80	5.46	1.34
6	8.62	8.25	0.37	7.00	6.50	0.5
7	5.58	5.48	0.10	4.50	4.28	0.22
8	10.87	10.15	0.72	9.60	9.25	0.35
Mean	7.96	7.40	0.56	6.65	6.01	0.65
SD	1.89	1.72	0.38	1.90	1.90	0.37

a ^a^ Modified Hausdorff distance value between CT and PET before registration.

b ^b^ Modified Hausdorff distance value between CT and PET after global registration.

c ^c^ Difference value between a and b.

d ^d^ Modified Hausdorff distance value between CT and PET after GMI‐based demons registration.

e ^e^ Modified Hausdorff distance value between CT and PET after PMI diffeomorphic‐based demons registration.

f ^f^ Difference value between d and e.

SD=standard deviation.

## IV. DISCUSSION

In this work we evaluated and compared two deformable registration algorithms for PET/CT alignment in eight patients with esophageal cancer(s). The modified Hausdroff distance was used as a similarity metric to evaluate the registration accuracy of the two presented algorithms. The preliminary results showed that the PMI diffeomorphic‐based demons algorithm was better in aligning PET and CT images than the GMI‐based demons algorithm. That is probably because the diffeomorphic‐based demons combining a Lie group framework on diffeomorphisms and optimizing for Lie groups. So this algorithm ensures a smoother invertible transformation. Furthermore, the PMI diffeomorphic‐based demons algorithm allows estimation of mutual information on individual image points; that is to say, every pixel has its own contribution to the global mutual information and the global mutual information can be computed locally, while the GMI‐based demons algorithm is based on gradient of mutual information between two images. An accurate deformable registration framework for PET and CT alignment can be used for accurate positioning tumor target in radiotherapy treatment planning. Precise definition of tumor target can largely reduce toxicity to surrounding organs and tissues and ensure sufficient dosage to the tumor target in treatment planning therapy.^(14)^ Currently, in 3D image‐guide radiation therapy (IGRT) GTVs are usually manually delineated by oncologists in treatment planning system on the planning day. In order to exclude the effects caused by respiratory motion and other physiological factors, margins or standardized safety margins (SSMs) are often added to the GTVs for better lesion coverage. The SSMs are usually defined according to specific cancer category rather than patient‐specific, which potentially induce the risk of underdosing of tumor target and overdosing of surrounding normal tissues and organs. More accurate tumor target volume positioning can be realized by considering both structural information and functional metabolism information based on accurate registration of PET and CT. The differences in Hausdorff distance between before registration and after using PMI diffeomorphic‐based method in our study lie within 1–2 voxels. However, typical safety margins in radiation therapy lie within 5–10 mm, which will affect planning tumor volume (PTV) contouring, resulting in inaccurate radiation therapy.

In this study, we addressed the problem of assessing the performance of two multimodal registration algorithms based on the GMI and the PMI diffeomorphic, two previously published demons‐based algorithms in aligning PET and CT images for radiation therapy. In the work by Jin and colleagues,^(18)^ the authors evaluated the GMI‐based demons algorithm by comparing it with the MI‐based algorithm in aligning 10 pairs of PET and CT images. They chose the PET images as the reference image and the corresponding CT image as the floating image, which was different from our study. Since the authors meant to accurately position the tumor volume target in CT images based on accurate registration of PET and CT images, it might be more rational to register PET to CT. Comparing to the commonly used MI‐based deformable registration algorithm, the presented GMI‐based demons showed high efficiency in aligning PET and CT images evaluated by the modified Hausdorff distance, with the difference ratio improved up to 10%. In the study by Vercauteren et al.,^(16)^ the authors evaluated the accuracy of the proposed PMI diffeomorphic‐based demons algorithm by comparing it with free‐form (FFD) deformation registration method on artificially distorted images. The multimodal registration algorithm was tested on T1 and T2 MR images from the BrainWed MRI Simulated Normal Brain Database.^(22)^ The quality of the registration was evaluated by the RMS of displacement field, maximum distance error (MDE) and global mutual information values. The results showed that the PMI diffeomorphic‐based demons algorithm outperformed the FFD method in all distortion levels. The limitation of this study was that the PMI diffeomorphic‐based demons algorithm was not validated on real patient subjects. So in this study, we quantitatively evaluated and compared the two currently proposed algorithms in cancer patients for performing accurate cancer radiation therapy.

This pilot study included a limited number of patients and assessed only patients with esophageal cancer(s). A larger pool of patients is still needed in future studies to answer the following questions: 1.) Is this comparison effective to cancers in other locations treated by radiation therapy, such as liver or lung cancers? 2.) Since there is no established golden standard for evaluation, can we find a more reasonable and robust method to validate the accuracy of deformable registration algorithm? 3.) How does the resolution level affect the convergence of cost function since we only employed three levels for registration? It is obvious that the deformation of lung or liver can be more easily affected by the respiratory motion, compared with esophagus investigated in this study. So there might be different results if we used PET/CT images of lung or liver to evaluate the performance of the two presented deformable image registration algorithms, due to the suboptimal contrast of liver CT and the large deformation of lung PET/CT. In addition, we found that, for both algorithms, the registration accuracy was gradually improved and the convergence speed was gradually slowed with the increasing of the standard deviation (σ) of Gaussian kernel. However, after σ was greater than 2, both the convergent speed and the registration accuracy were much similar with the increased σ values. To balance the registration accuracy and the convergence speed, the parameters in this study were set according to the previous literatures.^17^, ^18^

## V. CONCLUSIONS

We have successfully evaluated and compared the GMI‐based demons algorithm and the PMI diffeomorphic‐based demons algorithm for PET and CT alignment in patients with esophageal cancer(s). Preliminary results demonstrated that the PMI diffeomorphic‐based demons algorithm showed better alignment of esophageal PET/ CT images compared with the GMI‐based demons algorithm, which could be helpful for positioning tumor target in radiation treatment planning.

However, the evaluation and comparison in the current study was limited by its small sample size. A larger follow‐up study has a better chance of quantifying the reliability of our results.

## ACKNOWLEDGMENTS

This work is supported by National Natural Science Foundation of China (No. 61201441), National Natural Science Foundation of China (81472811), and Natural Science Grant of Shandong Province (ZR2010HM010).

## Supporting information

Supplementary Material
