# Supplementary material for: Evaluation of GMI and PMI diffeomorphic‐based demons algorithms for aligning PET and CT Images
Source: J Appl Clin Med Phys. 2015 Jul 8;16(4):18–30. doi: 10.1120/jacmp.v16i4.5148 (PMC5690013; doi:10.1120/jacmp.v16i4.5148)
Supplement: Supplementary file 1 — Supplementary Material [file ACM2-16-018-s001.docx]

**Evaluation of GMI- and PMI Diffeomorphic- based Demons Algorithms for Aligning PET and CT Images**

**Juan Yang ^1^, Hongjun Wang ^1, *^, You Zhang ^2^, Yong Yin ^3^**

*^1^School of Information Science and Engineering, Shandong University, Jinan, Shandong, China, 250100*

*^2^ Medical Physics Program, Duke University, Duke University Medical Center, Durham, NC, USA, 27705*

*3 Department of Radiation Oncology, Shandong Cancer Hospital and Institute, Jinan, Shandong, China, 250100*

* Please send all correspondence and reprint request to

Hongjun Wang, Ph.D.

School of Information Science and Engineering

Shandong University,

Jinan, Shandong, China, 250100

Email: [*bright-4030@163.com*](mailto:bright-4030@163.com)

**Running title:** Deformable image registration for PET and CT images
